# A novel PET probe to selectively image heat shock protein 90α/β isoforms in the brain

**DOI:** 10.1186/s41181-024-00248-0

**Published:** 2024-03-04

**Authors:** Takayuki Sakai, Aya Ogata, Hiroshi Ikenuma, Takashi Yamada, Saori Hattori, Junichiro Abe, Shinichi Imamura, Masanori Ichise, Mari Tada, Akiyoshi Kakita, Hiroko Koyama, Masaaki Suzuki, Takashi Kato, Kengo Ito, Yasuyuki Kimura

**Affiliations:** 1 Department of Clinical and Experimental Neuroimaging, Center for Development of Advanced Medicine for Dementia, Research Institute, National Center for Geriatrics and Gerontology (NCGG), 7-430 Morioka-cho, Obu, Aichi 474-8511 Japan; 2 Department of Pharmacy, Faculty of Pharmacy, Gifu University of Medical Science (GUMS), Kani, Japan; 3 Department of Pathology, Brain Research Institute, Niigata University, Niigata, Japan; 4 Department of Chemistry and Biomolecular Science, Faculty of Engineering, Gifu University, Gifu, Japan

**Keywords:** HSP90, Heat shock protein, Positron emission tomography, Brain

## Abstract

**Background:**

Heat shock proteins (HSPs) are present throughout the brain. They function as molecular chaperones, meaning they help with the folding and unfolding of large protein complexes. These chaperones are vital in the development of neuropathological conditions such as Alzheimer’s disease and Lewy body disease, with HSP90, a specific subtype of HSP, playing a key role. Many studies have shown that drugs that inhibit HSP90 activity have beneficial effects in the neurodegenerative diseases. Therefore, HSP90 PET imaging ligand can be used effectively to study HSP90 in neurodegenerative diseases. Among four HSP90 isoforms, two cytosolic isoforms (HSP90α and HSP90β) thought to be involved in the structural homeostasis of the proteins related to the neurodegenerative diseases. Currently, no useful PET imaging ligands selectively targeting the two cytosolic isoforms of HSP90 have been available yet.

**Results:**

In this study, we developed a novel positron emission tomography (PET) imaging ligand, [^11^C]BIIB021, by ^11^C-radiolabeling (a positron emitter with a half-life of 20.4 min) 6-Chloro-9-[(4-methoxy-3,5-dimethylpyridin-2-yl)methyl]-9*H*-purin-2-amine (BIIB021), an inhibitor with a high affinity for and selectivity to HSP90α and HSP90β. [^11^C]BIIB021 was synthesized with a high yield, molar activity and radiochemical purity. [^11^C]BIIB021 showed a high binding affinity for rat brain homogenate as well as human recombinant HSP90α and HSP90β proteins. Radioactivity was well detected in the rat brain (SUV 1.4). It showed clear specific binding in PET imaging of healthy rats and autoradiography of healthy rat and human brain sections. Radiometabolite was detected in the brain, however, total distribution volume was well quantified using dual-input graphical model. Inhibition of p-glycoprotein increased brain radioactivity concentrations. However, total distribution volume values with and without p-glycoprotein inhibition were nearly the same.

**Conclusions:**

We have developed a new PET imaging agent, [^11^C]BIIB021, specifically targeting HSP90α/β. We have been successful in synthesizing [^11^C]BIIB021 and in vitro and in vivo imaging HSP90α/β. However, the quantification of HSP90α/β is complicated by the presence of radiometabolites in the brain and the potential to be a substrate for p-glycoprotein. Further efforts are needed to develop radioligand suitable for imaging of HSP90α/β.

**Supplementary Information:**

The online version contains supplementary material available at 10.1186/s41181-024-00248-0.

## Background

Neurodegenerative diseases are one of the health care challenges in the aging society. Neuropathological features in the two major neurodegenerative diseases, Alzheimer's disease (AD) and Lewy body disease (LBD) are characterized by aggregation of abnormal proteins, including both amyloid-β (senile plaques) and phosphorylated tau (neurofibrillary tangles) in AD, and α-synuclein (Lewy bodies) in LBD. Recently it has been found that ubiquitously found proteins in the brain, called molecular chaperones, play important functional roles in these neuropathological processes.

Molecular chaperones are proteins that assist the conformational folding or unfolding of large proteins or macromolecular protein complexes. There are many families of molecular chaperones depending on the way they assist in folding macro proteins. For example, one family called heat shock proteins (HSPs) become highly expressed in the experimentally induced heat stress. When tissue cells are exposed to stresses such as heat shock, oxidative stress, and inflammation, regenerated intracellular proteins may become structurally misfolded and accumulated as abnormal proteins. Molecular chaperones such as heat shock proteins are activated by these stresses and form a protein complex by combination of chaperones and other proteins (chaperome) to enhance their functions (Lackie et al. 2017). In response to stressful conditions, chaperomes can either restore or decompose misfolded proteins, acting as the first line of defense against stress (Voth and Jakob 2017). Due to its high metabolic demands, the central nervous system (CNS) is exposed to high stresses including the oxidative stress. For example, CNS is particularly vulnerable to the effects of aging. One of aging effects is an imbalance between chaperon and neuronal activity levels, resulting in an inability to restore/decompose misfolded proteins and subsequently leading to toxic protein accumulation and the development of neurodegenerative diseases. (Bohush et al. 2019; Wang et al. 2018).

Of many HSP subtypes, HSP90 has been shown to play a central role in the pathophysiology of neurodegenerative disease. Recently, HSP90 has become therapeutic targets for neurodegenerative diseases, because it is involved in the structural homeostasis of proteins including amyloid-β, tau, and α-synuclein (Alam et al. 2017). HSP90 inhibition studies have shown that the amount of tau aggregates and α-synuclein-induced toxicity are reduced in AD and LBD model rats, respectively (Luo et al. 2007; Auluck et al. 2002). In the stressed brain, the expression of HSP90 is expected to be increased. However, HSP90 levels in vitro appear complex and inconsistent between AD and LBD brains. In AD brains, HSP90 levels are paradoxically decreased by ~ 20–30%, whereas in LBD brains, HSP90 levels are moderately increased in visual inspection of Western blots (exact amount of the increase was not quantified) (Koopman and Rüdiger 2020; Uryu et al. 2006). The reason(s) for these discrepant in vitro findings is currently not well understood. However, considering the potential beneficial effects of drugs that inhibit HSP90 activity in these neurodegenerative diseases, the availability of PET imaging ligands targeting HSP90 may allow us to track changes in HSP90 levels in these diseases and they could also be an additional ligand to the arsenal currently used for pathophysiological studies of neurodegenerative diseases in general.

**Fig. 1:**
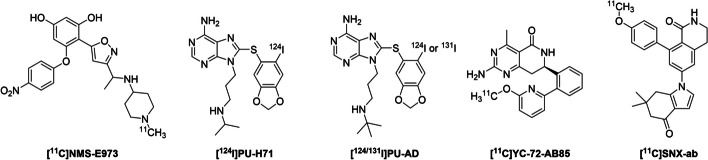
Structures of PET imaging ligands targeting HSP90

In fact, several Hsp90-targeting PET imaging ligands have already been reported (Vermeulen et al. 2019, 2021; Dunphy et al. 2020; Inda et al. 2020) (Fig. 1). These ligands have been developed based on the known HSP90 inhibitors. HSP90 has four isoforms: two isoforms (HSP90α and HSP90β) in the cytosol, one (TRAP1) in the mitochondria, and one (GRP94) in the endoplasmic reticulum (Li et al. 2020). HSP90α and HSP90β are mainly expressed in the cytoplasm and are involved in maintaining homeostasis of proteins, such as amyloid-β, tau and α-synuclein, by assisting the conformational changes of nascent polypeptides and refolding misfolded-proteins (Alam et al. 2017; Sanchez et al. 2020). Currently available PET ligands bind to all three subtypes of HSP90 isoforms except for [^11^C]SNX-ab (Cools et al. 2023). However this ligand failed to show sufficient brain uptake in mice (Cools et al. 2023).

**Scheme 1:**
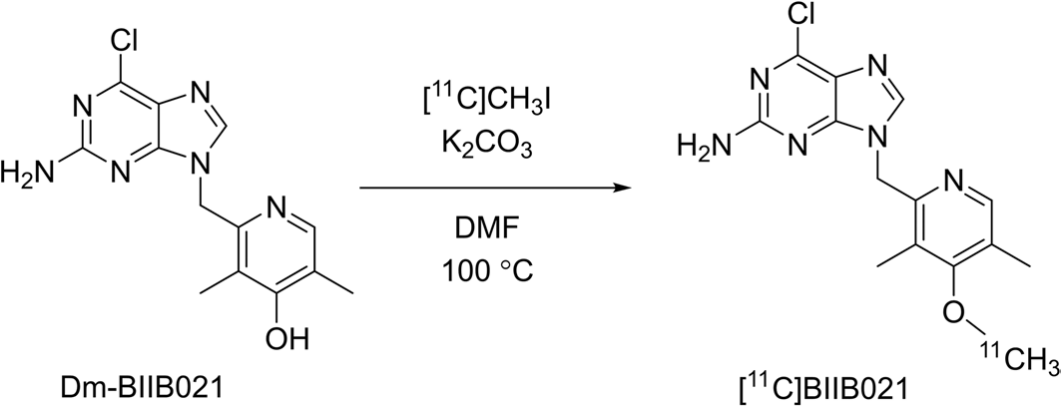
Radiosynthesis of [^11^C]BIIB021. Dm-BIIB021 was methylated with [^11^C]CH_3_I with K_2_CO_3_ in DMF at 100 °C for 10 min

In the current study, we report a novel PET imaging ligand [^11^C]BIIB021 (Scheme 1) to selectively measure HSP90α/β in the brain of patients with neurodegenerative diseases. As a lead compound for the synthesis of HSP90α/β-selective PET imaging ligand, we focused on a different HSP90α/β specific inhibitor than one used for the previously described study (Kasibhatla et al. 2007) namely BIIB021, a moderately selective purine-based compound (*K*_i_ = 2 nM for HSP90α, 4 nM for HSP90β, 176 nM for GRP94, and 62 nM for TRAP1) (Ernst et al. 2014). BIIB021 has a methoxy group on the pyridine ring, allowing ^11^C-labeling at this site. When calculated using ChemDraw® Professional version 16.0.1.4(77), [^11^C]BIIB021 is expected to have good brain uptake, considering its structure with two hydrogen donors, seven hydrogen acceptors, and clogP of 1.9. [^11^C]BIIB021 has a molecular weight of 318 considered to be suitable for brain PET imaging, although its polar surface area is slightly larger than optimal at 92 Å^2^. Considering these features, we decided to synthesize an ^11^C-labeled PET imaging ligand selective to HSP90α/β, [^11^C]BIIB021, and evaluate its usefulness in vitro and in vivo.

## Methods

### Chemicals and synthesis

We synthesized BIIB021 and Dm-BIIB021 as the standard and precursor compounds for [^11^C]BIIB021, respectively, following methodology described by Kasibhatla et al. (Additional file 1: Supplementary Scheme S1, Kasibhatla et al. 2007). Detailed procedures are found in the supplementary information.

### Preparation of [^11^C]BIIB021

Radioactive ^11^C was generated by the ^14^N (p, α) ^11^C nuclear reaction using a cyclotron (CYPRIS HM-18, Sumitomo Heavy Industries, Co. Ltd, Japan). ^11^C-methylation of **5** to [^11^C]BIIB021, HPLC purification and formulation were achieved automatically using specially designed equipment (Sumitomo Heavy Industries, Co. Ltd, Japan) The [^11^C]iodomethane obtained was trapped in 250 µL of anhydrous DMF containing 0.5–5 mg of **5** (1.64–16.4 µmol) and 10 mg of potassium carbonate (2.3 µmol) at −15 °C to −20 °C, and then the reaction mixture was heated to 100 °C for 10 min. The radioactive mixture containing [^11^C]BIIB021 was diluted with 1 mL of HPLC mobile phase, and transferred onto a column (10 mm I.D. × 250 mm, CAPCELL PAK C_18_, SHISEIDO, Tokyo, Japan) attached to the JACSO HPLC system. Elution with 20:80 v/v acetonitrile /2 M ammonium formate (in sterile water) at a flow rate of 5 mL/min gave a radioactive fraction corresponding to pure [^11^C]BIIB021 (Additional file 1: Supplementary Figure S1, retention time: 7.6 min). The fraction was collected in a rotary evaporator with 25% ascorbic acid solution 50 µL and evaporated to dryness at about 75 °C under reduced pressure. The residue was dissolved in 3 mL of sterile tween-saline, and filtered through a 0.22 µm Millex^Ⓡ^-GV filter (Merck, Darmstadt, Germany). At the end of synthesis, 1.43–2.94 GBq of [^11^C]BIIB021 was obtained with the molar activity of 52.6–144.1 GBq/µmol. The calculation of the molar activity of [^11^C]BIIB021 was performed using the HPLC calibration curve shown in (Additional file 1: Supplementary FigureS3).

### Measurement of logP of BIIB021

We measured the logP value of BIIB021 using its carbon-11 labeled version, [^11^C]BIIB021. We added a small amount of [^11^C]BIIB021 (50 MBq, 2–6 µL) to four vials, each containing equal volumes of 1-octanol (2 mL) and PBS (2 mL, pH 7.4, at 19 °C). We mixed the contents by vortexing for 3 min, followed by centrifuging them at 4500 × g for 5 min at 19 °C. After centrifugation, we took 500 µL samples from both the octanol and PBS layers in each vial and measured their radioactivity with a gamma counter (AccuFlex γ7001, Aloka Co., Ltd., Tokyo, Japan). We then calculated the logP values based on the radioactivity ratios in the octanol and PBS layers.

### Animals

F344/NSlc rats (male, 8–10 week old, Japan SLC, Inc., Hamamatsu, Japan) and a wild type TgF344-19 rat (male, 14 month old, obtained from Rat Resource and Research Center, MO, USA and bred inhouse) (Cohen et al. 2013) were used for the current study. The animals used here were maintained and handled in accordance with the National Research Council's Guide for the Care and Use of Laboratory Animals and our institutional guidelines. Protocols for the present animal experiments were approved by the Animal Ethics Committee of the National Center for Gelotology and Geriatrics.

### Binding assays of rat brain homogenate

Frozen brain powder derived from a wild type TgF344-19 rat (14-month-old) were homogenized in 50 mM Tris–HCl buffer, pH 7.4 (at 25 °C), containing protease inhibitor cocktail (cOmplete™, EDTA-free; Roche). The homogenate were incubated with 0.3–40 nM [^11^C]BIIB021 (molar activity: 144.1 GBq/μmol) in the absence or presence of unlabelled BIIB021 at 10 μM in Tris–HCl buffer, pH 7.4 (at 4 °C), for 30 min at 30 °C. Non-specific binding of [^11^C]BIIB021 was determined in the presence of 10 μM BIIB021. The dissociation constant (*K*_D_) was estimated with a one-site total binding model performed using GraphPad Prism (version 9 for Mac, GraphPad Software, www.graphpad.com).

### Binding assays of human recombinant HSP90α and β protein

Human recombinant HSP90α and β protein with a His-tag (20 pmol BPS Bioscience, USA) were separately mixed with μMACS Anti-His Tag MicroBeads (50 μL, Miltenyi Biotec, USA) and incubated at 0 °C for 30 min (total volume was 400 μL). Then, the mixture was incubated with 0.35–45 nM [^11^C]BIIB021 (molar activity: 99.4 or 102.8 GBq/μmol, respectively) with or without unlabeled BIIB021 in the reaction buffer (20 mM HEPES, pH 7.4, 5 mM MgCl_2_, 50 mM KCl, 20 mM Na_2_MoO_4_, 500 μL) at 30 °C for 30 min. Each reaction solution was applied to μMACS columns (Multi-8 columns, Miltenyi Biotec, USA) placed in a magnetic field of the MultiMACS M96 Separator (Miltenyi Biotec, USA) and washed three times with the buffer (100 μL × 2, 500 μL × 1). Then, the buffer (500 μL) was applied to the μMACS columns placed outside the magnetic field. Radioactivity of the elution was measured with a gamma counter (AccuFLEX γ7001, ALOKA Co., Ltd. Tokyo, Japan). The free concentration of BIIB021 was corrected to account for ligand depletion based on the radioactivity in the flow-through liquid after applying the reaction solution to the columns. *K*_D_ values were calculated in the same manner as for the rat brain homogenate binding assay.

### Off-target protein binding assays

Off-target protein binding screens were conducted by Sekisui Medical Inc. Binding inhibition effects of 10 µM BIIB021 were evaluated in competitive radioligand assays against 60 commonly expressed proteins including neurotransmitter receptors, ion channels, and transporters in the brain. Percent inhibition for each protein was calculated following the reported method (Okamura et al. 2013).

### PET imaging

To investigate in vivo specific binding of [^11^C]BIIB021, baseline and blocking PET studies were performed on F344/NSlc rats (male, 8–10 weeks old). Rats were scanned for 120 min on a small animal PET scanner (FX3200, TriFoil Imaging, CA, USA) after an injection of [^11^C]BIIB021 (15–60 MBq, equivalent to 0.4–0.8 nmol) via the tail vein under the isoflurane anesthesia (~ 2.0%). In these blocking PET studies, BIIB021 (2.0 mg/kg) or NVP-HSP990 (2.5 mg/kg, Selleck Chemicals, Houston, TX, USA) were intravenously injected to a rat as a blocking agent 30 min before the injection of [^11^C]BIIB021.

All PET images were reconstructed with the three-dimensional ordered subset expectation maximization method (4 subsets and 20 iterations; voxel size: 0.6 × 0.5 × 0.5 mm with the resolution of 0.92 mm full width at half maximum at the center of view). Time-activity curves were generated using data extracted from PET images. A template of preset volumes of interest was applied to the PET images to extract time-activity curves for the following ROIs: whole brain, amygdala, striatum, cingulate, thalamus, hippocampus, and cerebellum. All image analyses were performed using PMOD version 4.3 (PMOD Technologies Ltd., Zurich, Switzerland).

### Blood analysis during PET imaging

In these baseline PET studies, arterial blood sampling was performed on three rats through a catheter inserted into the femoral artery. The sampling protocol included continuous sampling for the first 4 min (12 samples taken every 15 s and 2 samples taken every 30 s), followed by intermittent sampling at specified time points post-injection (5, 7, 10, 15, 30, and 60 min). Radioactivity concentrations in the plasma and whole blood were measured for each sample using a gamma counter (AccuFLEX γ7001, Aloka Co., Ltd. Tokyo, Japan). Cross calibration was performed beforehand of the PET scanner and gamma counter against a standard well scintillation counter (IGC-8, Aloka Co., Ltd. Tokyo, Japan).

### Metabolite analysis in the rat blood and brains

For blood and brain metabolite analyses, F344/NSlc rats (10 weeks old) were intravenously injected via the tail vein with [^11^C]BIIB021 (210–270 MBq, equivalent to 1.7–5.1 nmol) under isoflurane anesthesia (~ 2.0%). Arterial blood was sampled four times from the rats at 5, 15, 30, and 60 min. The blood samples were centrifuged (12,000 rpm, 3 min, 4 °C) to separate out plasma. The supernatant (0.10 mL) was resuspended in acetonitrile (0.10 mL), and the mixture was placed on ice for 3 min after inverted mixing and deproteinized by centrifugation (12,000 rpm, 3 min, 4 °C).

Rats were sacrificed by decapitation immediately after the blood sampling was completed. A brain hemisphere was homogenized in radio-immunoprecipitation assay (RIPA) buffer (4.0 mL, Fujifilm Wako Pure Chemical Corporation, Osaka, Japan) on ice. The homogenate was centrifuged (12,000 rpm, 3 min, 4 °C), and the supernatant (about 3.0 mL) was collected, resuspended in acetonitrile (3.0 mL) on ice, and deproteinized by centrifugation (12,000 rpm, 3 min, 4 °C).

The supernatants, plasma and brain, were injected into a radio-HPLC (Prominence LC-20 system, Shimazu, Kyoto, Japan and FC-4100, Eckert & Ziegler Radiopharma, Hopkinton, MA, USA) and analyzed by an ODS-4 column (Cadenza CD-C18, 3 μm, 10 mm I.D. × 150 mm, Imtakt, Kyoto, Japan) with a gradient of acetonitrile/0.1% trifluoroacetic acid buffer (from 15/85 to 45/55) a at a flow rate of 3.0 mL/min. The unmetabolized fraction was calculated as the ratio of unmetabolized [^11^C]BIIB021 peak area to the total areas.

### PET quantification analysis in rats

A dual-input graphical model (Ichise et al. 1999; Kimura et al. 2015) was chosen for the quantification, where the effects of radiometabolites in the brain are taken into account. In this model, apparent total distribution volume (denoted as α) is the slope of the graphical plot, and it represents the sum of the distribution volumes of specific binding and non-specific binding of both the parent and radiometabolites. The brain time activity curves were corrected for cerebral blood volume (5%).  

### Efflux transporter

[^11^C]BIIB021 can be a substrate of efflux transporters. To investigate this possibility, [^11^C]BIIB021 PET imaging (36 MBq, equivalent to 1.2 nmol) was performed in a rat (10 weeks old) with pre-administration (60 min) of a p-glycoprotein inhibitor, tariquidar (7.5 mg/kg, Cayman Chemical, Ann Arbor, MI, USA). Quantification was performed as above.

### Autoradiography

A wild-type F344Tg rat (14-month-old) brain was removed rapidly and then frozen immediately in powdered dry ice. Postmortem healthy human brains were obtained from autopsies carried out at the Brain Research Institute, Niigata University on a subject with colon cancer with liver metastases but without any significant brain pathologies. In vitro autoradiography was performed using 20-μm thick fresh frozen sagittal sections for rat whole brain and 7-μm-thick for the frontal section of the human subject. Sections were first pre-incubated only in bovine serum for 30 min and then incubated in bovine serum containing [^11^C]BIIB021 (25–47 nM) at 30 °C for 30 min without or with BIIB021 (10 µM) or NVP-HSP990 (10 µM, Selleck Chemicals, Houston, TX, USA) as a blocking agent. The samples were then rinsed with the serum (4 °C) twice for 2 min each, and dipped into water (4 °C) for 10 s. The sections were subsequently dried by treating with cold air and were then exposed to an imaging plate (BAS-SR2040, Fuji Film, Tokyo, Japan). The imaging plate was scanned with a Typhoon FLA9500 (GE Healthcare, IL, USA) to acquire autoradiograms.

### Statistics

Results are expressed as the mean ± standard deviation of experiments conducted multiple times. ..

## Results

### Radiosynthesis

[^11^C]BIIB021 was synthesized by reacting Dm-BIIB021 with [^11^C]iodomethane under a basic condition (Scheme 1). Initially, we found that [^11^C]BIIB021 was partially radiolytically degraded in the post-preparative HPLC process, and radiochemical purity was about 72%. Therefore, we decided to add 50 μL of a 25% ascorbic acid aqueous solution as a radical scavenger before removing the fraction at reduced pressure. The radiochemical purity was improved to > 99%. (16 ± 2.0% radiochemical yield, molar activity of 150 ± 26 GBq/µmol at the end of radiosyntheses, n = 5) The chemical identity of [^11^C]BIIB021 was confirmed to have the identical HPLC retention time with that of the standard compound (Additional file 1: Supplementary Figure S2).

### Measurement of logP

The logP value was 1.6 ± 0.1, which was close to the calculated value of 1.9.

### Binding assays of rat brain homogenate and human recombinant HSP90α and β protein

**Fig. 2:**
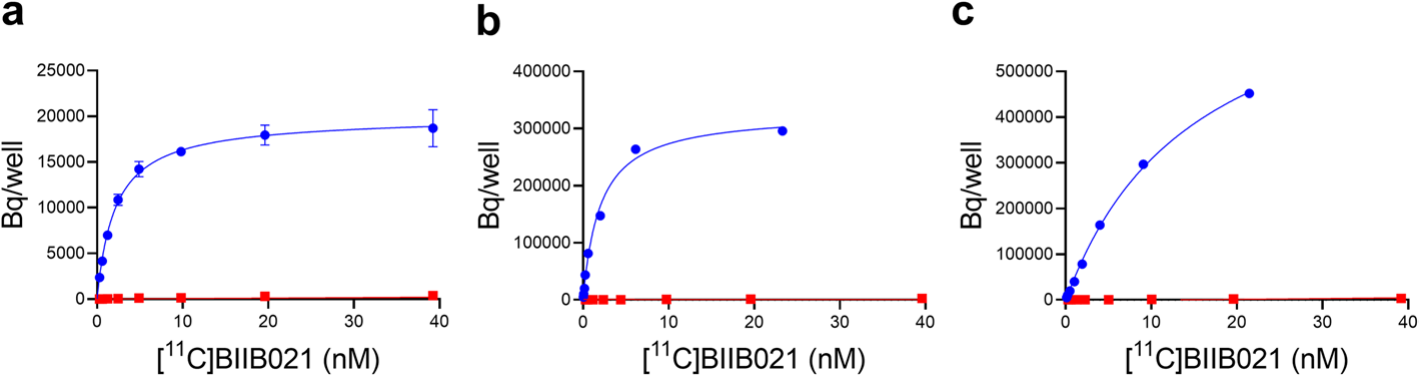
Binding of [^11^C]BIIB021 in rat brain homogenates **a** and human recombinant HSP90α **b** and HSP90β proteins **c**. Total (blue circles) and non-specific (red squares) binding of [^11^C]BIIB021 to a healthy rat brain homogenate, HSP90α and HSP90β. Homologous blocking was performed by 10 μM of nonlabelled BIIB021. The binding by BIIB021 was described by a 1-site model and fitted curves are indicated

To evaluate the binding affinity of [^11^C]BIIB021, a homologous binding assay was performed using brain homogenate of a healthy rat and human recombinant HSP90α and HSP90β protein. The curve fitting of the total binding gave a stable estimation of *K*_D_ value of 2.1 nM for rat brain homogenates (Fig. 2a). The stable estimated *K*_D_ values were 2.0 nM for HSP90α and 15 nM for HSP90β, indicating that [^11^C]BIIB021 preferably targets HSP90α compared to HSP90β (Fig. 2b, c). The previously reported affinity measurements were *K*_i_ = 2 nM for HSP90α, 4 nM for HSP90β, 176 nM for GRP94, and 62 nM for TRAP1 (Ernst et al. 2014). Thus, [^11^C]BIIB021 appears to selectively bind to HSP90α and HSP90β isoforms.

To evaluate the possibility of off-target protein binding of BIIB021, we studied 60 other commonly expressed proteins in the brain. Of these, melatonin receptor 1 showed the highest inhibition rate at ~ 26% inhibition at 10 μM BIIB021 (Additional file 1: Supplementary Table S1). Thus, BIIB021 does not appeasers to bind other protein than HSP90α and HSP90β.

### PET imaging

**Fig. 3:**
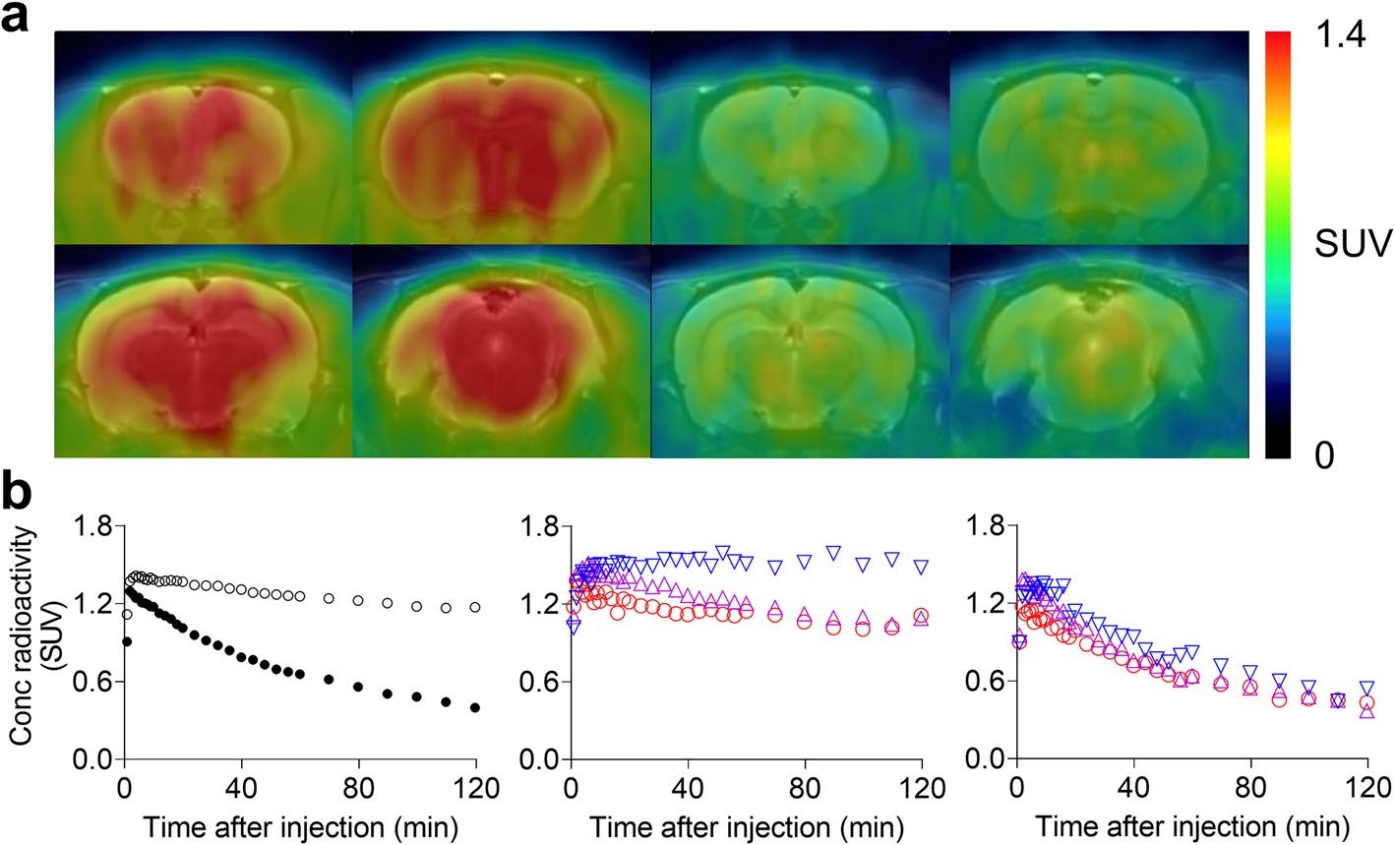
PET imaging of [^11^C]BIIB021 in healthy rats. **a** Brain PET images of rats injected [^11^C]BIIB021 with or without nonlabelled BIIB021. Images are averaged from 0 to 120 min after injection. Coronal section images are displayed with color scale units in standardized uptake values overlayed on a template MR image. Left four images are at baseline and right four images are after blocking with 2 mg/kg BIIB021. **b** Whole brain (left) and regional time-activity curves at the baseline (middle) and block experiments (right). In the left graph, increased washout was observed after blocking with 2 mg/kg BIIB021 (filled circle) compared to at the baseline (open circle). In the middle and right graph, regional differences seen at the baseline (middle) became smaller after the blocking (right). Regions: amygdala (red circle), cerebellum (purple triangle), and thalamus (blue inverted triangle)

PET imaging was conducted in two healthy rats after intravenous administration of [^11^C]BIIB021 in baseline and blocking experiments with pre-administration of the standard compound (2 mg/kg BIIB021) or another HSP90 selective inhibitor (2.5 mg/kg NVP-HSP990). In the baseline condition, brain radioactivity peaked in 4 min (SUV 1.4), followed by a slow washout (Fig. 3a left, 3b left and middle). Radioactivity was the highest in the thalamus and relatively low in the cerebellum, amygdala, and hippocampus. In the blocking condition with pre administration of the standard compound, the time-activity curves showed a faster washout and smaller regional radioactivity differences indicating the presence of specific binding in the whole brain (Fig. 3a right, b left and right). Pre-administration of NVP-HSP990 also showed similar blocking effects on the time-activity curves supporting the evidence that [^11^C]BIIB021 specifically binds to HSP90 (Additional file 1: Supplementary Figure S4).

### Metabolite analysis in rat blood and brain

**Fig. 4:**
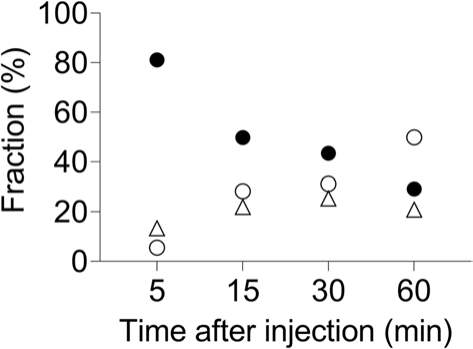
Average fractions of radioactivity from unchanged [^11^C]BIIB021 (filled circle), its major radiometabolite (open circle) and minor radiometabolites (open triangle) in rat plasma

One major radiometabolite and some inseparable minor ones were detected (Fig. 4 and Additional file 1: Supplementary Figure S5). At 60 min after injection, ~ 30% of the radioactivity was from unmetabolized. These radiometabolites were more hydrophilic than the parent. The same radio metabolites were found in the brain homogenate. At ~ 60 min after injection, ~ 50% of the radioactivity in the brain was from unmetabolized (Additional file 1: Supplementary Figure S5).

### Brain kinetics analysis

**Fig. 5:**
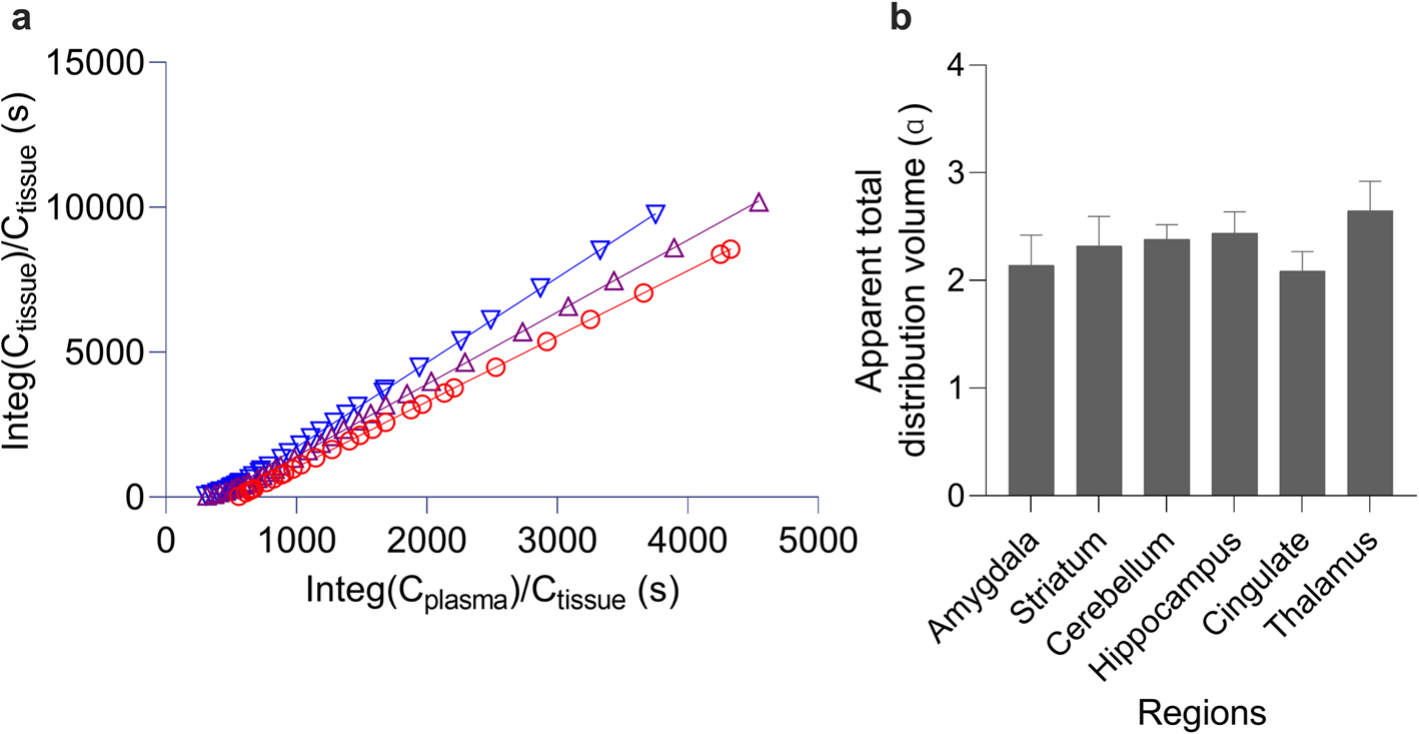
Quantification of [^11^C]BIIB021 PET in rats. **a** Representative dual-input graphical plots for the amygdala (red circle), cerebellum (purple triangle), and thalamus (blue inverted triangle). These plots became linear after t* = 10 min providing a stable estimation of apparent total distribution volumes. **b** Regional apparent total distribution volumes estimated using the dual input graphical model

In consideration of the presence of brain-penetrable radioactive metabolites, we employed a dual-input graphical model to quantify total distribution volumes of [^11^C]BIIB021 in the brain as a measure of radioligand binding to HSP90 from the time-activity curves in the brain and plasma. The apparent total distribution volumes were well identified as slops of graphical plots (Fig. 5a). The thalamus showed the highest apparent total distribution volume, while the cingulate showed the lowest (Fig. 5b). Inhibition of p-glycoprotein increased brain radioactivity concentrations suggesting that this radioligand may be a p-glycoprotein substrate. However, apparent total distribution volume values with and without p-glycoprotein inhibition were nearly the same (α = 2.3, Additional file 1: Supplementary figure S6).

### Autoradiography

**Fig. 6:**
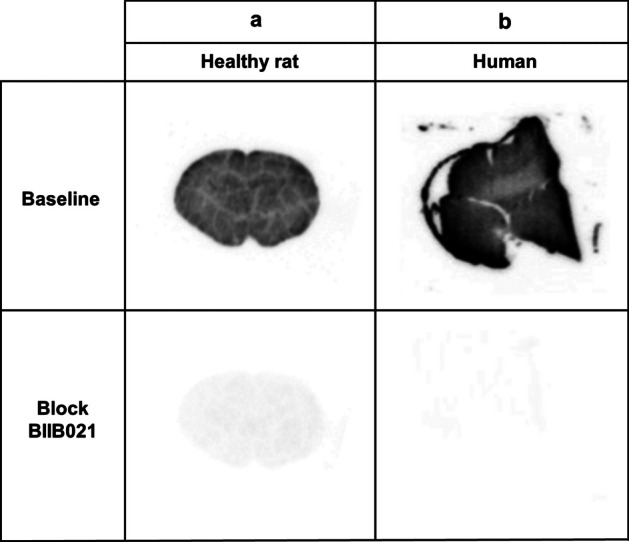
Autoradiography of coronal brain sections of healthy rats **a** and frontal sections of healthy humans **b** with and without nonlabeled BIIB021 (10 μM) as a blocking agent

Autoradiography was performed on brain sections of a healthy rat and human (Fig. 6). Radioactivity accumulated in the brain with higher activity in the gray matter and lower activity in the white matter of the rat brain section and human frontal section. That radioactivity accumulation was blocked with addition of non-labeled BIIB021 indicating the presence of specific binding in the healthy rat and human brains. Addition of NVP-HSP990 instead of BIIB021 also showed similar blocking effects supporting the evidence that [^11^C]BIIB021 specifically binds to HSP90 (Additional file 1: Supplementary Figure S7).

## Discussion

In this study, we developed a novel PET imaging ligand, [^11^C]BIIB021, selectively targeting HSP90α and HSP90β by radiolabeling BIIB021, an inhibitor with a high affinity for and selectivity to HSP90α and β. [^11^C]BIIB021 was synthesized with a high yield, molar activity, and radiochemical purity. [^11^C]BIIB021 showed a high binding affinity for rat brain homogenate as well as human recombinant HSP90α and HSP90β proteins. This radioligand was well taken up into the rat brain (SUV 1.4) and showed clear specific binding in PET imaging of healthy rats and autoradiography of healthy rat and human brain sections. The radioligand kinetics were well described by the dual-input graphical model that accounts for the effects of radiometabolite in the brain.

### Radiosynthesis

We synthesized [^11^C]BIIB021 according to the method of Kasibhatla et al.(Kasibhatla et al. 2007). The standard and precursor compounds, BIIB021 and Dm-BIIB021 respectively, were obtained in a moderately high yield as reported previously (Kasibhatla et al. 2007). Then, we successfully synthesized [^11^C]BIIB021 by [^11^C]methylation of the phenolic hydroxyl group in Dm-BIIB021 under basic conditions by adding potassium carbonate and stabilized with the addition of a radical scavenger to prevent radiolysis.

### In vitro and vivo studies

In the binding assays, we confirmed the high binding affinity of [^11^C]BIIB021 for HSP90α and HSP90β in binding assays, showing the *K*_D_ values of 2.0 nM for human recombinant HSP90α and 15 nM for HSP90β. The higher binding affinity of this ligand for HSP90α than HSP90β makes [^11^C]BIIB021 advantageous for PET imaging because it is the HSP90α that increases more significantly by stress than does HSP90β (Maiti and Picard 2022). Although we did not confirm this ligand’s affinity for other HSP90 isoforms in this study, other studies suggested that this ligand does not have any significant affinity for other HSP90 isoforms (Ernst et al. 2014). Additionally, we did confirm that this ligand does not have any significantly high affinity for other commonly present proteins in the brain.

In the autoradiography, we confirmed specific binding of this tracer in both rat and human brain sections by using two HSP90 selective inhibitors, BIIB021 itself and NVP-HSP990. Its uptake distribution was consistent with the known distribution of HSP90α, where this protein is mainly expressed in the gray matter but also in the white matter to a lesser extent (Sidera et al. 2004).

In the PET imaging, we observed specific binding in the rat brain by pre-administration of two HSP90 selective inhibitors, BIIB021 itself and NVP-HSP990. However, gray/white matter distinction in the rat brain was unclear due to limited spatial resolution of our small animal PET system. In human PET studies, considering this ligand’s good brain uptake, we expect to visualize regional distribution of HSP90α/β more clearly.

In the radiometabolite analysis, we found entry of radiometabolites in the brain. However, the dual-input graphical model that takes radio-metabolites entering the brain into account allows estimation of the apparent total distribution volume, the ratio of brain radioactivity and plasma parent concentration at equilibrium. This apparent distribution volume represents a sum of specific and nonspecific distribution volumes of both the parent and metabolites. Metabolites of BIIB021 have been reported previously (Xu et al. 2013) and are likely to have a negligibly low affinity for HSP90 compared to BIIB021 based on its structure-activity relationship study (Kasibhatla et al. 2007).

Inhibition of p-glycoprotein increased brain radioactivity concentrations suggesting that this radioligand may be a p-glycoprotein substrate. However, apparent total distribution volume values with and without p-glycoprotein inhibition were nearly the same. Further studies are warranted to investigate the potential effects of p-glycoprotein on the quantification of HSP90α/β with this radioligand.

## Conclusion

We developed a novel PET imaging ligand [^11^C]BIIB021 selectively targeting HSP90α/β. We have been successful in synthesizing [^11^C]BIIB021 and in vitro and in vivo imaging HSP90. Our data suggest that radiometabolite of this radioligand enter the brain, and this ligand may be substrate of p-glycoprotein, complicating the quantification of HSP90α/β. Based on this knowledge, further efforts are needed to develop radioligand suitable for imaging of HSP90α/β.

### Supplementary Information

**Additional file 1.** Supplementary methods, results (including Scheme S1), figures (S1–S7), and a table (S1).

## Data Availability

The datasets generated and/or analyzed during the current study are available from the corresponding author on reasonable request.

## Abbreviations

AD: Alzheimer's disease
*B*_max_: Available receptor site concentration
CNS: Central nervous system
HPLC: High performance liquid chromatography
HSPs: Heat shock proteins
I.D: Internal diameter
*K*_D_: Equilibrium dissociation constant
LBD: Lewy body disease
NMR: Nuclear magnetic resonance
PET: Positron emission tomography
ppm: Parts per million
SUV: Standardized uptake value
UV: Ultraviolet
*V*_T_: Distribution volume
v/v: Volume per volume

## References

[CR1] Alam Q, Alam MZ, Wali Sait KH, Anfinan N, Noorwali AW, Kamal MA (2017). Translational shift of HSP90 as a novel therapeutic target from cancer to neurodegenerative disorders: an emerging trend in the cure of Alzheimer’s and Parkinson’s diseases. Curr Drug Metab.

[CR2] Auluck PK, Chan HYE, Trojanowski JQ, Lee VMY, Bonini NM (2002). Chaperone suppression of α-synuclein toxicity in a Drosophila model for Parkinson’s disease. Science.

[CR3] Bohush A, Bieganowski P, Filipek A (2019). Hsp90 and its co-chaperones in neurodegenerative diseases. Int J Mol Sci.

[CR4] Cohen RM, Rezai-Zadeh K, Weitz TM, Rentsendorj A, Gate D, Spivak I (2013). A transgenic alzheimer rat with plaques, tau pathology, behavioral impairment, oligomeric Aβ, and frank neuronal loss. J Neurosci.

[CR5] Cools R, Vermeulen K, Narykina V, Leitao RCF, Bormans G (2023). Radiosynthesis and preclinical evaluation of [^11^C]SNX-ab as an Hsp90α, β isoform-selective PET probe for in vivo brain and tumour imaging. EJNMMI Radiopharm Chem.

[CR6] Dunphy MPS, Pressl C, Pillarsetty N, Grkovski M, Modi S, Jhaveri K (2020). First-in-Human Trial of Epichaperome-Targeted PET in Patients with Cancer. Clin Cancer Res.

[CR7] Ernst JT, Liu M, Zuccola H, Neubert T, Beaumont K, Turnbull A (2014). Correlation between chemotype-dependent binding conformations of HSP90α/β and isoform selectivity-Implications for the structure-based design of HSP90α/β selective inhibitors for treating neurodegenerative diseases. Bioorganic Med Chem Lett [internet].

[CR8] Ichise M, Fujita M, Seibyl JP, Paul N, Verhoeff LG, Baldwin RM (1999). Graphical analysis and simplified quantification of striatal and extrastriatal dopamine D2 receptor binding with [^123^I]epidepride SPECT. J Nucl Med.

[CR9] Inda MC, Joshi S, Wang T, Bolaender A, Gandu S, Koren J (2020). The epichaperome is a mediator of toxic hippocampal stress and leads to protein connectivity-based dysfunction. Nat Commun.

[CR10] Kasibhatla SR, Hong K, Biamonte MA, Busch DJ, Karjian PL, Sensintaffar JL (2007). Rationally designed high-affinity 2-amino-6-halopurine heat shock protein 90 inhibitors that exhibit potent antitumor activity. J Med Chem.

[CR11] Kimura Y, Ichise M, Ito H, Shimada H, Ikoma Y, Seki C (2015). PET quantification of tau pathology in human brain with ^11^C-PBB3. J Nucl Med.

[CR12] Koopman MB, Rüdiger SGD (2020). Alzheimer cells on their way to derailment show selective changes in protein quality control network. Front Mol Biosci.

[CR13] Lackie RE, Maciejewski A, Ostapchenko VG, Marques-Lopes J, Choy WY, Duennwald ML (2017). The Hsp70/Hsp90 chaperone machinery in neurodegenerative diseases. Front Neurosci.

[CR14] Li L, Wang L, You Q-D, Xu X-L (2020). Heat Shock Protein 90 Inhibitors: An Update on Achievements, Challenges, and Future. J Med Chem [internet].

[CR15] Luo W, Dou F, Rodina A, Chip S, Kim J, Zhao Q (2007). Roles of heat-shock protein 90 in maintaining and facilitating the neurodegenerative phenotype in tauopathies. Proc Natl Acad Sci U S A.

[CR16] Maiti S, Picard D (2022). Cytosolic Hsp90 isoform-specific functions and clinical significance. Biomolecules.

[CR17] Okamura N, Furumoto S, Harada R, Tago T, Yoshikawa T, Fodero-Tavoletti M (2013). Novel ^18^F-labeled arylquinoline derivatives for noninvasive imaging of Tau pathology in Alzheimer disease. J Nucl Med.

[CR18] Sanchez J, Carter RT, Cohen SM, Blagg SJB (2020). Old and new approaches to target the Hsp90 chaperone [Internet]. Curr Cancer Drug Targets.

[CR19] Sidera K, Samiotaki M, Yfanti E, Panayotou G, Patsavoudi E (2004). Involvement of cell surface HSP90 in cell migration reveals a novel role in the developing nervous system. J Biol Chem [internet].

[CR20] Uryu K, Richter-Landsberg C, Welch W, Sun E, Goldbaum O, Norris EH (2006). Convergence of heat shock protein 90 with ubiquitin in filamentous α-synuclein inclusions of α-synucleinopathies. Am J Pathol [internet].

[CR21] Vermeulen K, Naus E, Ahamed M, Attili B, Siemons M, Luyten K (2019). Evaluation of [^11^C]NMS-E973 as a PET tracer for in vivo visualisation of HSP90. Theranostics.

[CR22] Vermeulen K, Cools R, Briard E, Auberson Y, Schoepfer J, Koole M (2021). Preclinical Evaluation of [^11^C]YC-72-AB85 for in Vivo Visualization of Heat Shock Protein 90 in Brain and Cancer with Positron Emission Tomography. ACS Chem Neurosci.

[CR23] Voth W, Jakob U (2017). Stress-Activated Chaperones: A First Line of Defense. Trends Biochem Sci.

[CR24] Wang K, Shang Y, Dou F (2018). Brain aging: Hsp90 and neurodegenerative diseases. Adv Exp Med Biol.

[CR25] Xu L, Woodward C, Dai J, Prakash C (2013). Metabolism and excretion of 6-chloro-9-(4-methoxy-3, 5-dimethylpyridin-2- ylmethyl)-9H-purin-2-ylamine, an HSP90 inhibitor, in rats and dogs and assessment of its metabolic profile in plasma of humans. Drug Metab Dispos.

