# Supplementary material for: A novel PET probe to selectively image heat shock protein 90α/β isoforms in the brain
Source: EJNMMI Radiopharm Chem. 2024 Mar 4;9:19. doi: 10.1186/s41181-024-00248-0 (PMC10912062; doi:10.1186/s41181-024-00248-0)
Supplement: Supplementary file 1 — Additional file 1. Supplementary methods, results (including Scheme S1), figures (S1–S7), and a table (S1). [file 41181_2024_248_MOESM1_ESM.docx]

Supplementary information

A novel PET probe to selectively image heat shock protein 90α/β isoforms in the brain

Takayuki Sakai^1^, Aya Ogata^1,2^, Hiroshi Ikenuma^1^, Takashi Yamada^1^, Saori Hattori^1^, Junichiro Abe^1^, Shinichi Imamura^1^, Masanori Ichise^1^, Mari Tada^3^, Akiyoshi Kakita^3^, Hiroko Koyama^4^, Masaaki Suzuki^4^, Takashi Kato^1^, Kengo Ito^1^, Yasuyuki Kimura*^1^

1. Department of Clinical and Experimental Neuroimaging, Center for Development of Advanced Medicine for Dementia, National Center for Geriatrics and Gerontology (NCGG), Obu, Japan
2. Department of Pharmaceutical, Faculty of Pharmacy, Gifu University of Medical Science (GUMS), Kani, Japan
3. Department of Pathology, Brain Research Institute, Niigata University, Niigata, Japan
4. Department of Chemistry and Biomolecular Science, Faculty of Engineering, Gifu University, Gifu, Japan

# First Author:

Takayuki Sakai, MSc, PhD
Department of Clinical and Experimental Neuroimaging, Center for Development of Advanced Medicine for Dementia, National Center for Geriatrics and Gerontology

7-430 Morioka-cho, Obu, Aichi 474-8511, Japan

TEL: +81562462311, FAX: +81562446596E-mail: tsakai@ncgg.go.jp

# Address correspondence and reprint requests:

Yasuyuki Kimura, MD, PhD
Department of Clinical and Experimental Neuroimaging, Center for Development of Advanced Medicine for Dementia, National Center for Geriatrics and Gerontology

7-430 Morioka-cho, Obu, Aichi 474-8511, Japan

TEL: +81562462311, FAX: +81562446596

E-mail: [yazkim@ncgg.go.jp](mailto:yazkim@ncgg.go.jp)

**Table of Contents**

| Topic | Display item(s) | page |
| --- | --- | --- |
| **Supplementary methods and results** |  | S3–6 |
| Chemicals |  | S3–4 |
| Synthesis |  | S4–6 |
| Synthesis of standard (BIIB021) and precursor (Dm-BIIB021) compounds. | Scheme S1 | S4 |
| **Supplementary figures and table** |  | S7–15 |
| Representative semi-preparative HPLC chromatogram of the [^11^C]BIIB021 fraction | Figure S1 | S7 |
| Representative analytical HPLC chromatogram of the formulated [^11^C]BIIB021 | Figure S2 | S8 |
| Calibration curve for the molar activity of [^11^C] BIIB021 | Figure S3 | S9 |
| Brain PET images of rats injected [^11^C]BIIB021 with or without pre-administration of NVP-HSP990. | Figure S4 | S10 |
| Representative HPLC charts of radiometabolite analysis in the plasma and brain | Figure S5 | S11 |
| PET imaging of [^11^C]BIIB021 with p-glycoprotein inhibition in a rat. | Figure S6 | S12 |
| Autoradiography of coronal brain sections of healthy rats and frontal sections of healthy humans with and without NVP-HSP990 | Figure S7 | S13 |
| Inhibitory effect of BIIB021 to various receptors, ion channels and transporters | Table S1 | S14–15 |
| **Reference** |  | S16 |

## **Supplementary methods and results**

## **Chemicals.**

Methanol were HPLC grade from Merck (Darmstadt, Germany). Citric acid monohydrate and sodium acetate from Kanto Chemical (Tokyo, Japan), ethylenediaminetetraacetic acid (EDTA) from Dojindo (Kumamoto, Japan), and Sodium octane sulfonate (SOS) from FUJIFILM Wako Pure Chemical (Osaka, Japan). All other chemicals were of analytical grade and were used without any further pretreatment.

General synthetic procedure

All commercially available reagents and solvents were used without further purification. Normal-phase thin layer chromatography (TLC) was carried out on Silica gel 60 F254 (Merck, 1.05715.0009) using reagent grade solvents. TLC was detected by the absorption of UV light (254 nm) or using a visualization reagent (molybdophosphoric acid). Column chromatography was performed on silica gel (Silica Gel 60, Kanto Chemical co., Inc. Tokyo, Japan) with mixed solvents as described. ^1^H and ^13^C NMR spectra were obtained for samples in the indicated solution at 25 ℃ utilizing the JNM-ECA500 spectrometer (JEOL. Ltd. Tokyo, Japan) at 500 MHz frequency for ^1^H or the JNM-AL400 spectrometer at 400 MHz frequency for ^1^H in CD_3_OD or deuterated dimethylsulfoxide (DMSO)-*d*_6_ with tetramethylsilane as an internal standard. ^1^H NMR chemical shifts are reported in terms of the chemical shift (δ, ppm) relative to the singlet corresponding to tetramethylsilane at 0 ppm. Splitting patterns are designated as follows: s, singlet; d, doublet; t, triplet; q, quartet; m, multiplet; br, broad. Coupling constants are reported in Hz. ^13^C NMR spectra were fully decoupled and are reported in terms of the chemical shift (δ, ppm) relative to a septet at δ = 39.5 ppm corresponding to DMSO-*d*_6_ or a septet at δ = 49.0 ppm corresponding to CD_3_OD. Electrospray ionization-mass (ESI)-mass spectrometry were carried out on LC-MS spectrometer (LCMS2020, Shimadzu Industrial Systems Co., Ltd.).

**Synthesis**

The synthesis of the standard and precursor compounds was conducted based on the method of Kasibhatla et al [1] (Scheme S1). The standard compound was obtained in a moderate yield (78%). The precursor compound was synthesized in two steps. First, methoxypyridine **1** was demethylated to afford hydroxypyridine **2** in a moderate yield (38％). Then the precursor compound Dm-BIIB021 was obtained in a moderate yield (44％) by the same reaction as the standard compound.


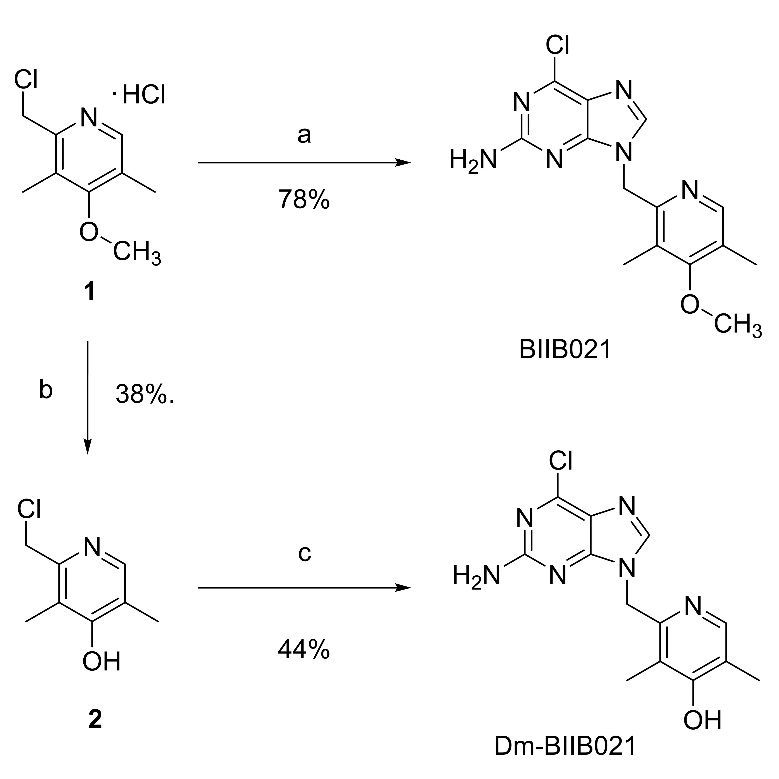


**Scheme S1.** Synthesis of standard (BIIB021) and precursor (Dm- BIIB021) compounds. (a) 2-amino-6-chloropurine, K_2_CO_3_, NaI, DMF, 40 °C, overnight; (b) Toluene, reflux, 19 h; (c) 2-amino-6-chloropurine, K_2_CO_3_, NaI, DMF, 40 °C, overnight.

**2-(chloromethyl)-3,5-dimethylpyridin-4-ol (2)** [1]**.**

2-(Chloromethyl)-4-methoxy-3,5-dimethylpyridine Hydrochloride (**1** ,1.11 g, 5 mmol) in toluene (0.9 mL) was stirred and refluxed under Ar. After 19 hours, the reaction mixture was cooled to room temperature and the solvent was removed under reduced pressure to give 2-(chloromethyl)-3,5-dimethylpyridin -4-ol (2) was obtained in 465 mg (54%). LRMS (ESI+): *m*/*z* [M+H]^+^: 186.

**6-Chloro-9-(4-methoxy-3,5-dimethylpyridin-2-ylmethyl)-9*H*-purin-2-ylamine (BIIB021)** [1]**.**

A suspension of 2-(Chloromethyl)-4-methoxy-3,5-dimethylpyridine Hydrochloride (**4**, 169.6 mg, 1.0 mmol), potassium carbonate (276 mg, 387 mmol), sodium iodide (15.0 mg, 0.1 mmol), and compound **2** (222.1 g, 1.0 mmol) in DMF (5 mL) was heated at 40 °C with stirring under Ar. After 15 h, the reaction mixture was cooled, and the inorganic solids were filtered and washed with DMF. Dilution with 10 mL of water induced the crystallization of the desired isomer **1** (BIIB021, 199.4 mg, 63%). Rf 0.20 (EtOAc); ^1^H NMR (DMSO-*d*_6_) δ = 8.09 (s, 1H), 8.03 (s, 1H), 6.85 (s, 2H), 5.37 (s, 2H), 3.75 (s, 3H), 2.31 (s, 3H), 2.17 (s, 3H).

**2-(2-Amino-6-chloropurin-9-ylmethyl)-3,5-dimethylpyridin-4-ol (Dm-BIIB021)** [1]**.**

A suspension of 2-amino-6-chloropurine **4** (459.5 mg, 2.71 mmol), potassium carbonate (1.12 g, 8.13 mmol), sodium iodide(40.6 mg, 0.27 mmol), and compound **3** (464.8 mg, 2.71 mmol) in DMF (5 mL) was heated at 40 °C with stirring under Ar. After 14 h, the reaction mixture was cooled, and the inorganic solids were filtered and washed with DMF. Dilution with 10 mL of water induced the crystallization of the desired isomer **5** (362.4 mg, 44%). ^1^H NMR (400 MHz, DMSO-*d*_6_) δ = 10.96 (s, 1H), 8.10 (s, 1H), 7.52 (s, 1H), 6.94 (s, 2H), 5.26 (s, 2H), 2.04 (s, 3H), 1.90 (s, 3H).

**Supplementary figures**


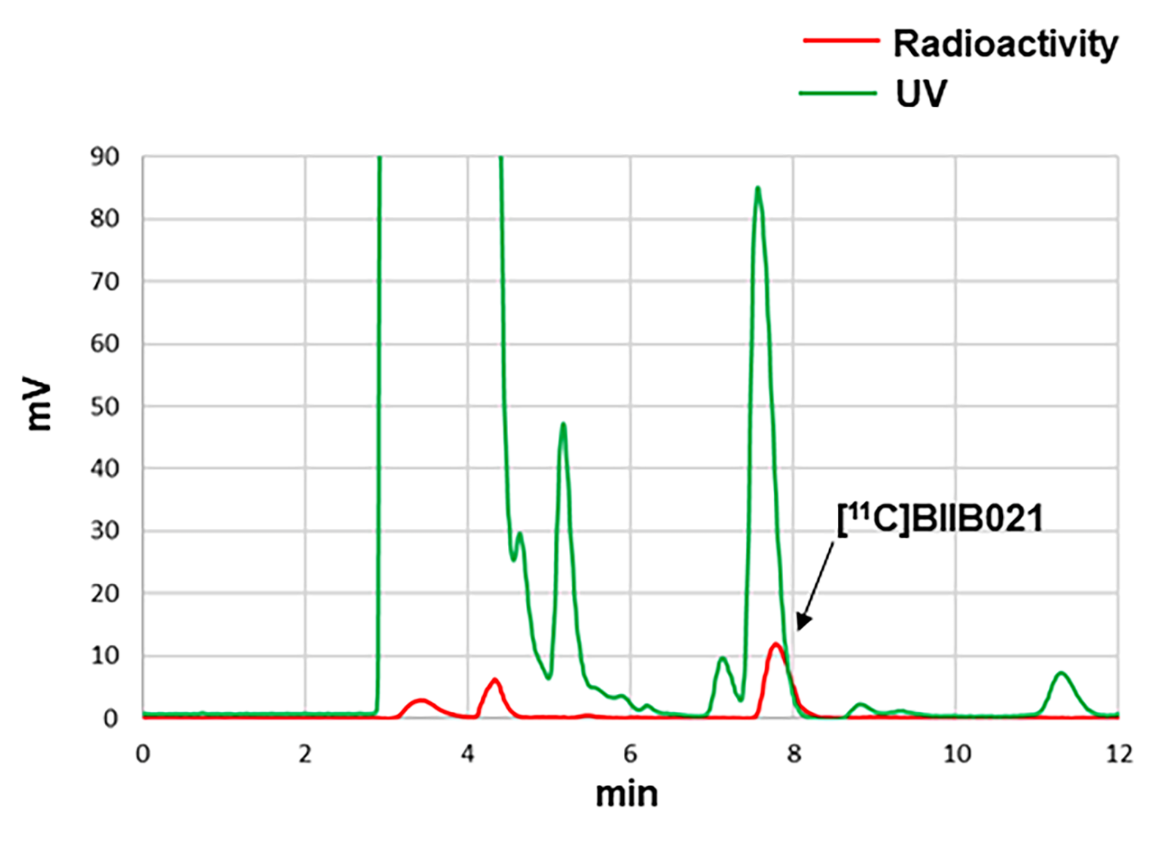


**Figure S1.** Representative semi-preparative HPLC chromatogram of the [^11^C]BIIB021 fraction. The semi-preparative HPLC was performed under the following conditions: preparative HPLC column: 10 mm I.D. × 250 mm, CAPCELL PAK C_18_, SHISEIDO, Tokyo, Japan; eluent: acetonitrile/0.2 M Ammonium formate (in sterile water) = 25:75 (*v*/*v*); flow rate: 5 mL/min; detection: ultraviolet (UV), 254 nm; retention time: 7.6 min. Note that retention peak times slightly differ between UV and RI because there is a small time gap between the UV and RI detectors.


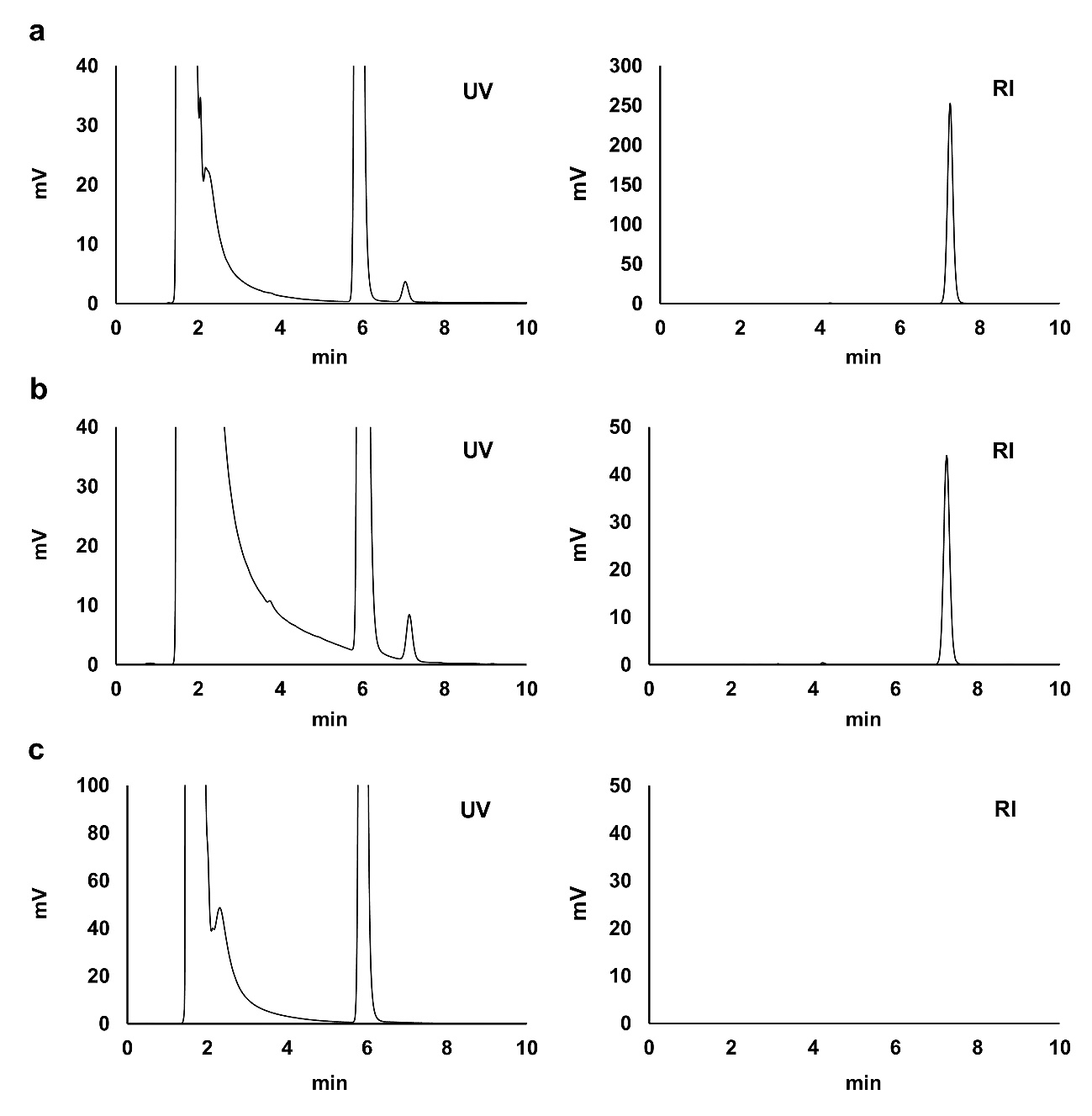


**Figure S2.** Representative analytical HPLC chromatogram of the formulated [^11^C]BIIB021 with ascorbic acid solution (UV and radioactivity (A)), the formulated [^11^C]BIIB021 with nonradioactive BIIB021 and ascorbic acid solution (UV and radioactivity (B)) and ascorbic acid solution. The analytical HPLC was performed under the following conditions: preparative HPLC column: CAPCELL PAK C18 UG 120 (5 μm, 4.6 mm i.d. × 250 mm, Osaka Soda CO., LTD., Osaka, Japan); eluent: acetonitrile/2 M Ammonium formate (in sterile water) = 25:75 (*v*/*v*); flow rate: 1 mL/min; detection: ultraviolet (UV), 254 nm; retention time: 7.2 min.

**
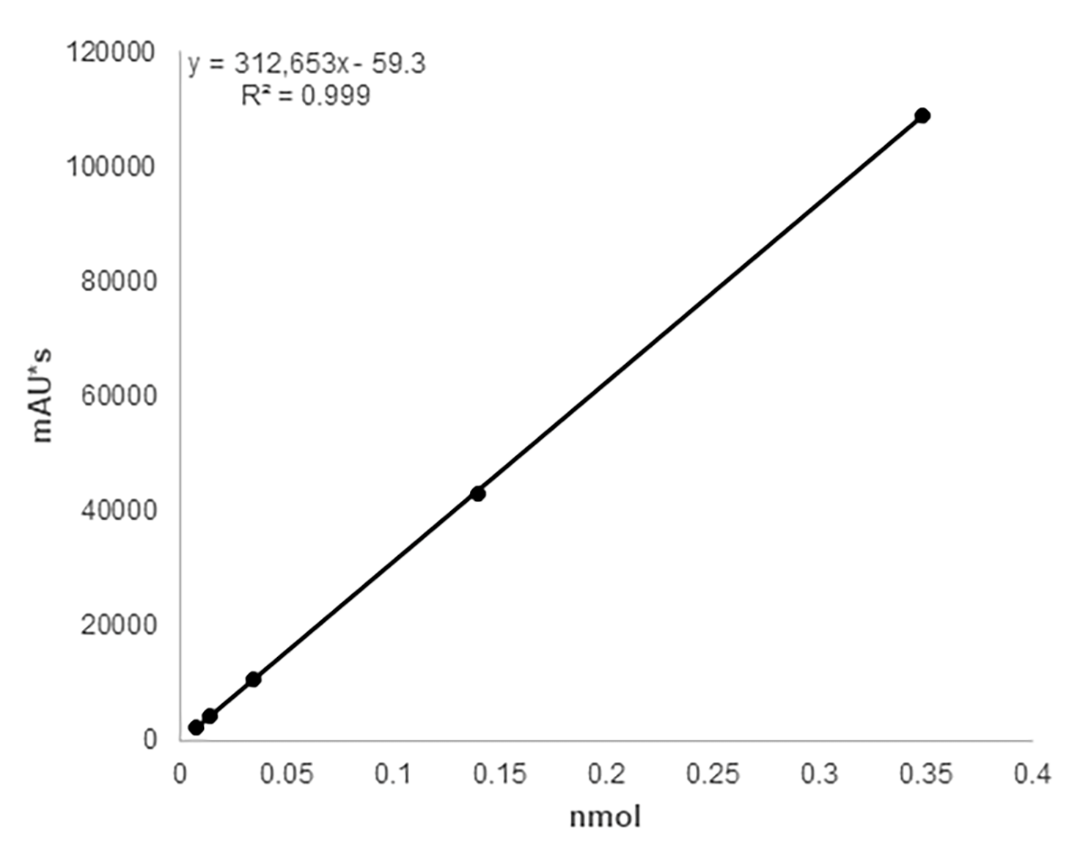
**

**Figure S3.** HPLC calibration curve to determine amount of BIIB021 used in the calculation of molar activity of [^11^C]BIIB021 in the range of 0.007–0.348 nmol. HPLC analysis was performed under the following conditions: Column: CAPCELL PAK C18 UG 120 (5 μm, 4.6 mm i.d. × 250 mm, Osaka Soda CO., LTD., Osaka, Japan); eluent: acetonitrile/0.2 M Ammonium formate (in sterile water) = 25:75 (*v*/*v*); flow rate: 1 mL/min; detection: UV, 254 nm; retention time, 7.2 min.


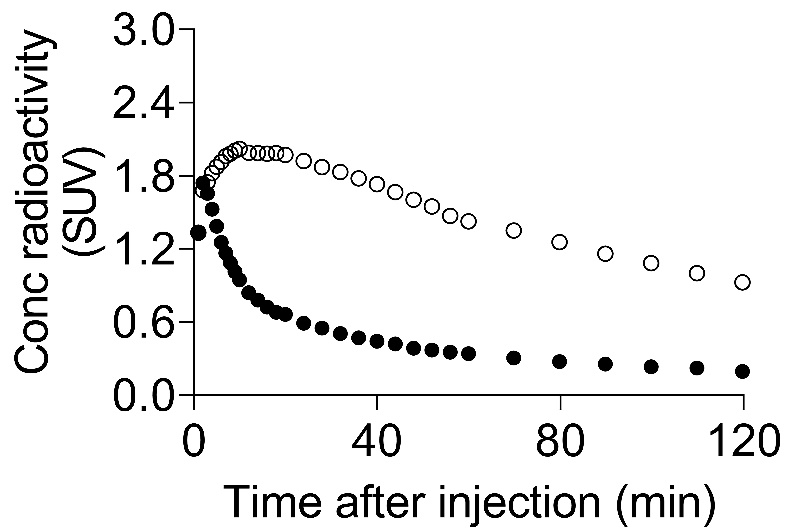


**Figure S4**. Brain PET images of rats injected [^11^C]BIIB021 with or without pre-administration of 2.5 mg/kg NVP-HSP990, which is structually different from BIIB021. Whole brain time-activity curves at the baseline (open circle) and block experiments (filled circle).


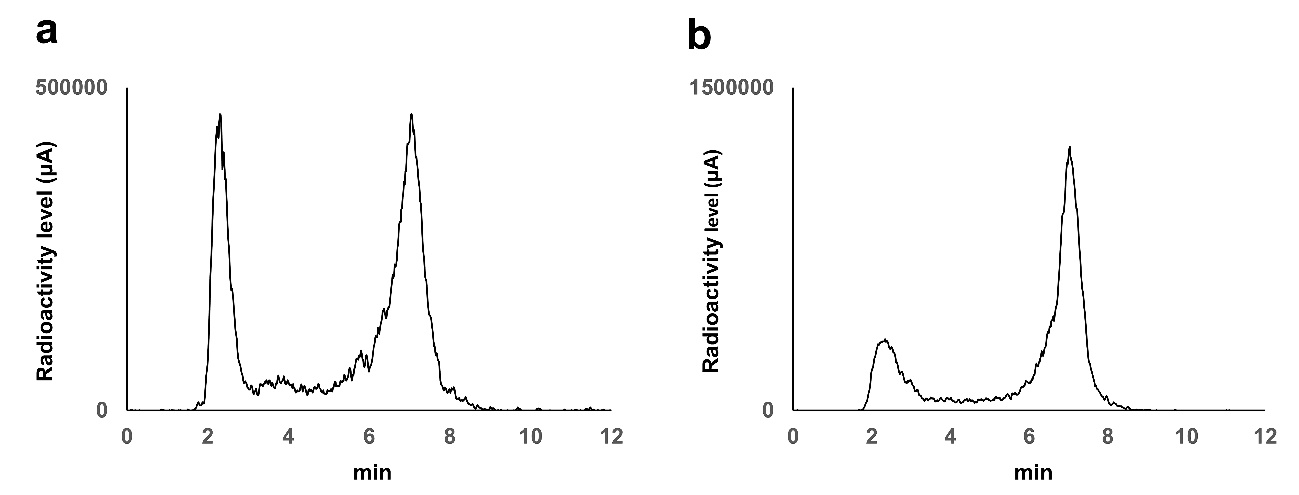


**Figure S5** . Representative HPLC charts of radiometabolite analysis in plasma (a) and the brain (b) at 60 min after injection. The parent, [^11^C]BIIB021, was detected at ~7 min retention time; a major radiometabolite at ~2.5 min, and minor inseparable radiometabolites between the two peaks.

**Figure S6**. PET imaging of [^11^C]BIIB021 with p-glycoprotein inhibition in a rat. (a) Whole brain time-activity curves of rats injected [^11^C]BIIB021 (b) Plasma time-activity curves The value at ~6 min is shown on both graphs, which differ in range of y-axis. (c) Apprent total distribution volume values in the whole brain at the baseline (n = 3) and after pre-administration of a p-glycoprotein inhibitor, tariquidar (7.5 mg/kg, n = 1).


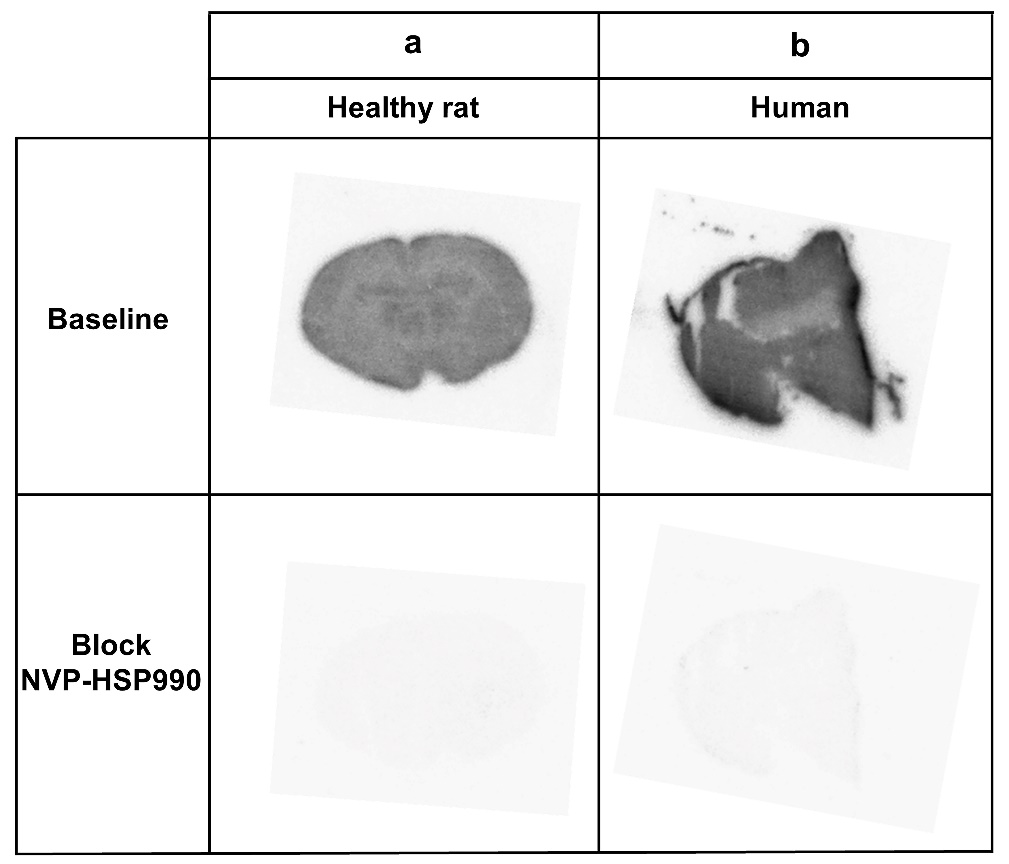


**Figure S7.** Autoradiography of coronal brain sections of healthy rats (a) and frontal sections of healthy humans (b) with and without NVP-HSP990 (10 μM) as a blocking agent.

**Table S1** Inhibitory effect of BIIB021 to various receptors, ion channels and transporters that are highly expressed in the brain.

| Assay system | Percentage of inhibition | | |  |
| --- | --- | --- | --- | --- |
|  | BIIB021  1×10^-5^ mol/L | Positive substance | | |
| Adenosine A1 (Human) | 8.4 | 100 | (DPCPX) | |
| Adenosine A2a (Human) | 14 | 100 | (CGS21680) | |
| α1A-Adrenergic | 1.8 | 99 | (Prazosin) | |
| α1B-Adrenergic | 0.0 | 100 | (Prazosin) | |
| α2A-Adrenergic (Human) | 13 | 100 | (Rauwolscine) | |
| α2B-Adrenergic (Human) | 0.0 | 100 | (Rauwolscine) | |
| β1-Adrenergic (Human) | 0.0 | 100 | ((±)-Propranolol) | |
| β2-Adrenergic (Human) | 0.9 | 100 | ((±)-Propranolol) | |
| Androgen | 0.7 | 99 | (Testosterone) | |
| Angiotensin AT1 (Human) | 0.0 | 100 | (Angiotensin II human) | |
| Bradykinin B1 (Human) | 0.0 | 100 | (Lys-(des-Arg^9^, Leu^8^)-  Bradykinin) | |
| Bradykinin B2 (Human) | 0.0 | 100 | (HOE140) | |
| Ca channel (Type L, Benzothiazepine) | 0.0 | 100 | ((+)-*cis*-Diltiazem) | |
| Ca channel (Type L, Dihydropyridine) | 0.0 | 100 | (Nitrendipine) | |
| Ca channel (Type N) | 2.3 | 100 | (ω-Conotoxin GVIA) | |
| Cannabinoid CB1 (Human) | 5.1 | 98 | ((*R*)-(+)-WIN55,212-2) | |
| Cannabinoid CB2 (Human) | 0.0 | 100 | ((*R*)-(+)-WIN55,212-2) | |
| CCK A (Human) | 2.8 | 100 | (CCK-8) | |
| Dopamine D1 (Human) | 16 | 99 | (*R*(+)-SCH-23390) | |
| Dopamine D2 short (Human) | 0.9 | 100 | ((+)-Butaclamol) | |
| Dopamine D3 (Human) | 0.4 | 100 | ((±)-7-OH-DPAT) | |
| Dopamine transporter (Human) | 1.1 | 100 | (GBR12909) | |
| Estrogen | 3.3 | 100 | (β-Estradiol) | |
| Endothelin ETA (Human) | 4.8 | 98 | (Endothelin-1 (Human)) | |
| Endothelin ETB (Human) | 4.7 | 95 | (Endothelin-1 (Human)) | |
| GABA A (Agonist site) | 2.7 | 100 | (Muscimol) | |
| GABA A (BZ central) | 2.5 | 100 | (Diazepam) | |
| GABA B | 0.0 | 100 | (GABA) | |
| GABA transporter | 2.0 | 100 | (GABA) | |
| Glucocorticoid (Human) | 0.0 | 96 | (Dexamethasone) | |

Continued

**Table S1** Inhibitory effect of BIIB021 to various receptors, ion channels and transporters that are highly expressed in the brain (Continued)

| Assay system | Percentage of inhibition | | | | |  |
| --- | --- | --- | --- | --- | --- | --- |
|  | BIIB021  1×10^-5^ mol/L | | Positive substance | | | |
| Glutamate (Kainate) | 4.6 | 100 | | (Kainic acid) |  |  |
| Glutamate (NMDA agonist site) | 1.8 | 99 | | (L-Glutamic acid) |  |  |
| Glutamate (NMDA glycine site) | 1.4 | 100 | | (MDL105,519) |  |  |
| Glutamate (NMDA phencyclidine site) | 0.7 | 97 | | ((+)-MK-801) |  |  |
| Histamine H1 (Human) | 8.1 | 100 | | (Pyrilamine) |  |  |
| Histamine H2 (Human) | 0.7 | 100 | | (Cimetidine) |  |  |
| Histamine H3 (Human) | 4.5 | 97 | | ((*R*)(−)-α-Methylhistamine) |  |  |
| Imidazoline (Central) | 0.0 | 98 | | (Guanabenz) |  |  |
| K channel KATP | 1.3 | 100 | | (Glybenclamide) |  |  |
| K channel SKCa | 16 | 99 | | (Apamin) |  |  |
| Melatonin MT1 (Human) | 26 | 100 | | (Melatonin) |  |  |
| Muscarinic M1 (Human) | 1.5 | 100 | | (Atropine) |  |  |
| Muscarinic M2 (Human) | 4.6 | 100 | | (Atropine) |  |  |
| Muscarinic M3 (Human) | 1.9 | 100 | | (Atropine) |  |  |
| Na channel Site 2 | 3.2 | 98 | | (Dibucaine) |  |  |
| Neurokinin NK1 (Human) | 6.2 | 100 | | (L-703,606) |  |  |
| Neuropeptide Y2 (Human) | 1.3 | 100 | | (Neuropeptide Y human) |  |  |
| Norepinephrine transporter  (Human) | 0.0 | 97 | | (Desipramine) |  |  |
| Nicotinic (Neuronal) | 0.5 | 99 | | ((±)-Nicotine) |  |  |
| Opiate δ (Human) | 4.2 | 100 | | (Naltriben) |  |  |
| Opiate κ (Human) | 14 | 100 | | (U-69593) |  |  |
| Opiate μ (Human) | 0.0 | 97 | | (DAMGO) |  |  |
| PAF | 6.9 | 98 | | (PAF) |  |  |
| Serotonin 5HT1A (Human) | 7.6 | 99 | | (Serotonin) |  |  |
| Serotonin 5HT2A (Human) | 4.9 | 100 | | (Ketanserin) |  |  |
| Serotonin 5HT3 (Human) | 18 | 100 | | (Tropisetron) |  |  |
| Serotonin transporter (Human) | 0.0 | 100 | | (Imipramine) |  |  |
| Sigma σ1 | 0.0 | 99 | | ((+)-Pentazocine) |  |  |
| Sigma σ2 | 2.4 | 95 | | (Haloperidol) |  |  |
| Vasopressin V1 | 3.0 | 100 | | ([Arg^8^]-Vasopressin) |  |  |

## **References**

1. Kasibhatla SR, Hong K, Biamonte MA, Busch DJ, Karjian PL, Sensintaffar JL, et al. Rationally Designed High-Affinity 2-Amino-6-halopurine Heat Shock Protein 90 Inhibitors That Exhibit Potent Antitumor Activity. J Med Chem. 2007;50:2767–78
